# Efficacy of pre-harvest weed control treatments on onion bulb storability

**DOI:** 10.1038/s41598-025-89970-6

**Published:** 2025-02-25

**Authors:** Karima F. Abdelgawad, Said A. Shehata, Ibrahim M. El-Metwally, Ebrahim R. El-Desoki, Kowthar G. El-Rokiek, Fathia A. Elkhawaga

**Affiliations:** 1 Vegetable Crops Department, Faculty of Agriculture, Cairo University, Giza, 12613 Egypt; 2 Botany Department, National Research Centre, Dokki, Cairo, 12622 Egypt

**Keywords:** Aqueous plant extracts, Orange peel proceeding waste, Mango leaves waste, Olive oil processing wastes

## Abstract

Storage experiments were carried out, in 2019 and 2020, to evaluate the effect of pre-harvest weed control treatments on quality attributes and storability of onion bulbs during a six-month storage at room temperature (25 ± 5 °C and 50–60% relative humidity) in corrugated paper boxes. Weed control treatments consisted of the aqueous extracts of orange peel processing waste (OPPW) 20%, mango leaves waste (MLW) 30%, and olive oil processing waste (OOPW) 30%, alone or mixed with half a dose of oxyfluorfen herbicide (938 ml ha^−1^), soil mulching with orange peel processing waste, mango leaves, olive oil processing waste, and rice straw (OPPWM, OOPWM, MLW, and RSM, respectively) at 10 tons ha^−1^, hoeing, oxyfluorfen herbicide (at 938 and 1875 ml ha^−1^), and an unweeded control treatment. OPPW mulch and hoeing treatments were the most effective treatments in improving storability, decreasing weight loss and decay percentages of onion bulbs as well as maintaining bulb quality attributes, *i.e.*, bulb firmness, dry matter, total soluble solids and total soluble sugars content during a six-months storage. It was concluded that using OPPW and MLW, as soil mulches or as aqueous extracts, for weed control within onion field, helps maintain onion bulb quality, and storability.

**Supplementary Information:**

The online version contains supplementary material available at 10.1038/s41598-025-89970-6.

## Introduction

Onion (*Allium cepa* L.) is an important vegetable crop that belongs to the Alliaceae family. During 2019 onion was cultivated on a total area of 87,948 hectares in Egypt, yielding 3,081,047 tons^1^. In 2019, 487 thousand tons of onion were exported, valued at almost 243 million dollars^1^. Shelf-life of onion bulb is a genetic trait, but it is also correlated with size, dry matter content, maturity degree at harvest and storage conditions of bulbs^2^. These features can be enhanced under adequate cultural practices and pre-harvest treatments including weed control^3^. Weed management is considered a serious problem in agricultural systems. Weeds grow with crops and compete with them for food, water and light, causing significant yield losses^4^. Onion crop has a weak competition potential with weeds as it slowly grows and can suffer from a successive flush of weeds. The onion plant also has narrow upright leaves that do not shade out to prevent weeds emerging in rows^5^. It has been reported that weed competition causes yield losses in onion bulbs varied from 55 to 72%^6–8^. Weed infestation has negative effects on the quality and storability of onion bulbs^6,9^, relative to the hand weeding treatment.

The use of herbicides and hoeing are the most common options for weed control in Egypt^10^. Hoeing has high economic cost and requires human labor that may not be available in many case^5^. While, the use of herbicides has harmful effects on the environment and is not permitted in some cases, such as organic farming^11^. Hence, there is a growing interest in using new natural products for managing weeds. Different plant extracts have been investigated as eco-friendly weed control treatments for many years. It has been found that the efficacy of natural extracts in weed inhibition is increased by the increase of its phenolic content^12^. This phenomenon is called allelopathy. Allelopathy is the ability of a plant to inhibit or stimulate the growth of another plant^13^. Recent studies reported that some agro-industrial wastes, such as orange peel, mango leaves and olive oil processing waste have allelopathic effects and could be used in weed control as aqueous extracts or as soil treatments^14–17^.

Several studies reported that pre-harvest weed control treatments had significantly decreased weight loss and maintained quality attributes during storage. Hassanein et al.^18^ reported that Amex herbicide + two hand hoeing treatment had significantly decreased weight loss of garlic bulb after a seven-month storage as compared with those obtained from unweeded treatment. Organic mulch treatments significantly reduced weight loss, splits, insect infested bulbs and rotten bulbs percentages of garlic, after a 150 day-storage period, compared to non-mulched treatments^19^. Different chemical and mechanical weed control treatments have been reported to decrease onion bulb weight loss and were higher in bulb diameter, total soluble solids percentage and dry matter during a six-month storage, compared with those obtained from unweeded treatment^9^. Therefore, this study was aimed to evaluate the effect of orange peel waste, mango leaves and olive oil processing waste, on quality attributes and storability of onion bulbs at ambient storage conditions.

## Results

### Weed control efficacy

**Fig. 1:**
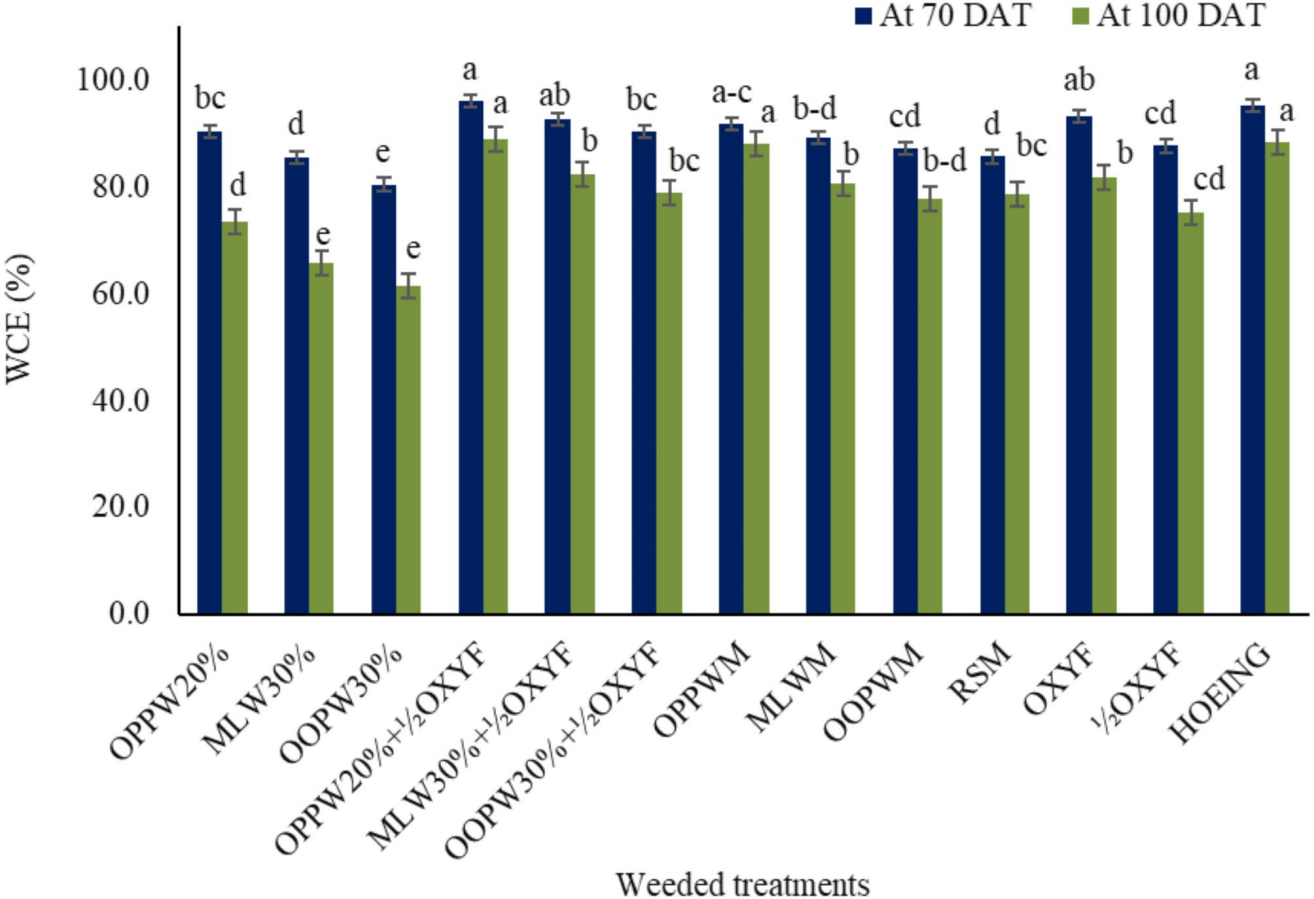
Weed control efficacy (WCE) % recorded at 70 and 100 days after transplanting (DAT) for different weed management methods. *Note* OPPW20%, MLW30%, and OOPW30% are the aqueous extracts of orange peel waste, mango leaves, and olive oil waste, respectively; OPPWM, MLWM, OOPWM and RSM are mulching with orange peel waste, mango leaves, olive oil waste, and rice straw, respectively; OXYF and ½OXYF are oxyfluorfen herbicide applied at rates of 1.8 and 0.9 l ha^ −1^, respectively. Dissimilar letters were significantly different at *p* < 0.05 according to the Duncan test. Error bars on the columns stands for ± standard deviation.

The onion field trials indicated that all weed control treatments significantly increased the weed control efficacy at 100 days after transplanting (DAT) compared to unweeded control (Fig. 1). The highest weed control efficacy at 70 DAT was recorded in the orange peel processing waste extract + ½dose of oxyfluorfen treatment (96.1%), without significant differences from that in the mango leaves waste extract + ½half dose of oxyfluorfen (91.6%), orange peel mulch (91.8%), and hoeing (95.2%) treatments (Fig. 1). The highest weed control efficacy at 100 DAT was found in the orange peel processing waste extract + ½dose of oxyfluorfen treatment (89%), hoeing (88.3%), and orange peel processing waste mulch (88%) treatments, without significant differences between them. The weed control efficacy of mango leaves waste extract + ½dose of oxyfluorfen (82.4%), olive oil processing waste extract + ½dose of oxyfluorfen (78.8%), mango leaves mulch (80.7%), olive oil waste mulch (77.9%), and rice straw mulch (78.8%) were not significantly different from that of the oxyfluorfen treatment (75.3%).

### Effect of weed control methods on weight loss and decay percentage of onion bulbs during storage

Results in Table 1 show a gradient increase in weight loss percentage during the storage period and the greatest losses occurred in the unweeded check during all storage periods. The most effective treatment in decreasing the weight loss percentage, during the whole storage period, was OPPW mulch treatment without significant difference from hoeing treatment at 3, 5 and 6 months from storage (Table 1).

**Table 1:** Effect of weed control methods on weight loss percentage of onion bulbs during storage.

Treatments	Storage period (month)					
	1	2	3	4	5	6
Orange peel waste AE^1^ 20%	3,1 e	5,9 ef	10,7 c	13,0 d	15,5 d	17,6 d
Mango leaves AE 30%	3,8 bc	6,9 c	12,2 b	14,2 c	16,6 c	19,3 b
Olive oil waste AE 30%	4,1 b	6,5 d	11,2 c	12,9 d	15,1 d	17,9 d
Orange peel AE 20% + ½oxyf^2^	2,3 f	4,7 g	7,6 f	9,2 h	11,5 g	14,7 g
Mango leaves AE 30% + ½oxyf	2,6 f	4,6 g	8,5 e	11,0 f	13,1 f	14,8 g
Olive oil waste AE 30% + ½oxyf	3,0 e	6,2 de	9,4 d	11,9 e	13,5 f	15,6 f
Orange peel waste mulch	1,0 h	3,5 i	6,2 g	8,0 i	11,0 h	13,3 h
Mango leaves mulch	3,3 de	6,0 ef	8,2 e	10,4 g	13,1 f	15,1fg
Olive oil waste mulch	3,6 cd	7,1 c	9,7ef	12,2 e	14,3 e	16,6 e
Rice straw mulch	3,0 e	5,7 f	9,9 d	12,2 e	14,1 e	17,9 d
Oxyfluorfen (1.8 l ha^−1^)^3^	2,3 f	6,4 d	8,5 e	12,2 e	14,4 e	16,6 e
Oxyfluorfen (0.9 l ha^−1^)^4^	3,9 bc	7,5 b	10,9 c	16,1 b	17,3 b	18,7 c
Hoeing (twice)	1,8 g	3,8 h	6,7 g	8,8 h	11,1 gh	13,4 h
Unweeded check	4,6 a	10,2 a	14,0 a	17,2 a	19,7 a	24,8 a

^1^AE, aqueous extract; ^2^oxyf, oxyfluorfen; ^3^ Oxyfluorfen (1.8 l ha^−1^): the recommended dose of the oxyfluorfen herbicide, ^4^oxyfluorfen (0.9 l ha^−1^): half of the recommended dose of the oxyfluorfen herbicide. Means followed by a letter in common in the same column are not significantly different at 0.05 level of probability according to Duncan multiple range test.

In most cases, there was an increase in decay percentage during the storage period. The lowest decay percentage at the end of the storage period was found in orange processing waste mulch treatment without significant difference from hoeing treatment at 1, 2, 4 and 6 months of storage (Table 2). Decay percentage was significantly decreased by the weed control treatments, compared to the decay in the unweeded check during the whole storage periods.

**Table 2:** Effect of weed control methods on decay percentage of onion bulbs during storage.

Treatments	Storage period (month)					
	1	2	3	4	5	6
Orange peel waste AE^1^ 20%	1,7 d	2,0 e	3,7 de	9,1 c–e	10,0 c–e	11,1 cd
Mango leaves AE 30%	1,7 d	2,6 d	5,1 b	10,0 b–d	11,1 b–d	12,6 bc
Olive oil waste AE 30%	2,0 bc	3,3 b	4,8 b	10,5bc	11,6 bc	12,6 bc
Orange peel AE 20% + ½oxyf ^2^	0,8 f	2,1 e	2,1 h	4,6 gh	5,4 f	7,4 f
Mango leaves AE 30% + ½oxyf	1,8 cd	2,6 d	3,6 de	8,7 c–e	9,5 de	9,8 de
Olive oil waste AE 30% + ½oxyf	2,1 b	3,1 bc	4,8 b	9,9 b–d	10,5 b–d	11,9 c
Orange peel waste mulch	0,4 g	0,9 g	2,5 g	2,5 i	3,2 g	4,1 g
Mango leaves mulch	0,8 f	2,5 d	3,5 de	6,0 fg	6,5 f	7,6 f
Olive oil waste mulch	1,0ef	2,7 d	4,1 c	7,3 ef	8,5 e	9,0 ef
Rice straw mulch	1,1 e	2,8 cd	3,9 cd	8,2 de	9,5 de	9,9 de
Oxyfluorfen (1.8 l ha^−1^)^3^	0,8 f	1,5 f	3,3 ef	7,9 d–f	9,5 de	9,2 d–f
Oxyfluorfen (0.9 l ha^−1^)^4^	2,1 b	3,1 bc	4,7 b	12,0 ab	12,0 b	13,9 b
Hoeing (twice)	0,5 g	1,1 g	3,0 f	3,7 hi	6,4 f	5,1 g
Unweeded check	2,7 a	4,5 a	6,7 a	13,3 a	14,0 a	23,4 a

^1^AE, aqueous extract; ^2^oxyf, oxyfluorfen; ^3^ Oxyfluorfen (1.8 l ha^−1^): the recommended dose of the oxyfluorfen herbicide, ^4^oxyfluorfen (0.9 l ha^−1^): half of the recommended dose of the oxyfluorfen herbicide. Means followed by a letter in common in the same column are not significantly different at 0.05 level of probability according to Duncan multiple range test.

### Effect of weed control methods on dry matter and firmness of onion bulbs during storage

All weed control treatments significantly increased onion bulb dry matter content compared to the unweeded check (Table 3). Dry matter of onion bulbs was increased by the prolongation of the storage period until 2 months then decreased till the end of the storage (Table 3). The highest dry matter percentage of onion bulb during the whole storage period was recorded in orange processing waste mulch followed by hoeing treatment at 2, 4 and 6 months of storage (Table 3).

**Table 3:** Effect of weed control methods on dry matter (%) of onion bulbs during.

Treatments	Storage period (month)			
	0	2	4	6
Orange peel waste AE^1^ 20%	11,2 cd	18,1 fg	17,0 f–h	16,6 ef
Mango leaves AE 30%	10,6 ef	17,7 g	16,8 gh	16,0 gh
Olive oil waste AE 30%	9,9 g	18,6 f	17,3 e–g	16,4 fg
Orange peel AE 20% + ½oxyf ^2^	11,0 cd	22,1 b	20,8 b	20,3 b
Mango leaves AE 30% + ½oxyf	11,2 cd	20,3 d	19,7 c	19,1 c
Olive oil waste AE 30% + ½oxyf	11,5bc	20,5 d	18,7d	17,1 e
Orange peel waste mulch	11,9 a	22,7 a	22,2 a	21,9 a
Mango leaves mulch	10,6 ef	21,4 c	19,4 c	17,8 d
Olive oil waste mulch	10,8 de	19,2 e	17,8 e	16,5 fg
Rice straw mulch	10,8 de	20,0 d	17,5 ef	15,7 h
Oxyfluorfen (1.8 l ha^−1^)^3^	10,6 ef	18,4 f	16,7 h	16,3 fg
Oxyfluorfen (0.9 l ha^−1^)^4^	10,2 fg	16,9 h	14,9 i	13,1 i
Hoeing (twice)	11,6 ab	21,9 bc	21,1 b	20,3 b
Unweeded check	9,0 h	14,5i	12,8 j	11,4 j

^1^AE, aqueous extract; ^2^oxyf, oxyfluorfen; ^3^ Oxyfluorfen (1.8 l ha^−1^): the recommended dose of the oxyfluorfen herbicide, ^4^oxyfluorfen (0.9 l ha^−1^): half of the recommended dose of the oxyfluorfen herbicide. Means followed by a letter in common in the same column are not significantly different at 0.05 level of probability according to Duncan multiple range test.

Weed control treatments showed higher values of onion bulb firmness compared to the unweeded check (Table 4). There was a reduction in onion bulb firmness by the prolongation of the storage period at room temperature for six months (Table 6). The highest values of onion bulb firmness, during the whole storage period, was found in orange processing waste mulch and hoeing treatments, without significant difference between them at 2, 4 and 6 months (Table 4).

**Table 4:** Effect of weed control methods on firmness (kg/cm^2^) of onion bulbs during storage.

Treatments	Storage period (month)			
	0	2	4	6
Orange peel waste AE^1^ 20%	12,5 ef	10,7 de	9,8 ef	9,6 gh
Mango leaves AE 30%	12,1 f	10,3 de	9,8 ef	9,4 gh
Olive oil waste AE 30%	11,0 g	10,2 e	9,6 f	9,2 h
Orange peel AE 20% + ½oxyf ^2^	13,9 c	12,7 b	11,6 b–d	11,2 c–e
Mango leaves AE 30% + ½oxyf	14,3 c	12,5 b	12,0 bc	11,5 b–d
Olive oil waste AE 30% + ½oxyf	13,0 de	11,3 cd	10,9 c–e	10,4 e–g
Orange peel waste mulch	16,0 a	15,2 a	13,9 a	13,2 a
Mango leaves mulch	13,7 cd	12,2 bc	11,3 b–d	10,8 d–f
Olive oil waste mulch	12,6 ef	11,3 cd	10,6 d–f	10,2 e–h
Rice straw mulch	13,6 cd	11,8 bc	10,7 d–f	10,3 e–g
Oxyfluorfen (1.8 l ha^−1^)^3^	13,7 cd	12,1 bc	12,1 b	11,8 bc
Oxyfluorfen (0.9 l ha^−1^)^4^	12,7 ef	11,3 cd	10,5 d–f	10,1 f–h
Hoeing (twice)	15,1 b	14,3a	13,2 a	12,4 ab
Unweeded check	10,1 h	8,5 f	8,0 g	7,6i

^1^AE, aqueous extract; ^2^oxyf, oxyfluorfen; ^3^ Oxyfluorfen (1.8 l ha^−1^): the recommended dose of the oxyfluorfen herbicide, ^4^oxyfluorfen (0.9 l ha^−1^): half of the recommended dose of the oxyfluorfen herbicide. Means followed by a letter in common in the same column are not significantly different at 0.05 level of probability according to Duncan multiple range test.

### Effect of weed control methods on total soluble solids and total soluble sugars of onion bulbs during storage

All the weeded treatments significantly increased total soluble solids content (TSS) of onion bulbs during the storage period compared to the unweeded check (Table 5). Total soluble solids increased by the prolongation of the storage period until 2 months then decreased till the end of the storage (Table 5). orange processing waste mulch and hoeing treatments were the most effective treatments in maintaining TSS content of onion bulbs without significant differences between them during all the storage periods (Table 5).

**Table 5:** Effect of weed control methods on total soluble solids (%) of onion bulbs during storage.

Treatments	Storage period (month)			
	0	2	4	6
Orange peel waste AE^1^ 20%	12,3 bc	12,9 c–e	11,3 e	9,7 f
Mango leaves AE 30%	12,2 b–d	12,8 c–e	11,4 e	9,9 f
Olive oil waste AE 30%	11,7 cd	12,7 c–e	11,4 e	10,4 e
Orange peel AE 20% + ½oxyf ^2^	12,3 bc	12,9 c–e	12,4 bc	11,5 c
Mango leaves AE 30% + ½oxyf	12,1 cd	13,2 bc	12,5 b	11,8 bc
Olive oil waste AE 30% + ½oxyf	12,1 cd	12,9 c–e	11,9 cd	11,5 c
Orange peel waste mulch	13,1 a	13,7 a	13,1 a	12,3 a
Mango leaves mulch	11,9 cd	13,0 cd	12,4 bc	10,8 d
Olive oil waste mulch	11,9 cd	12,7 c–e	12,3 bc	11,5 c
Rice straw mulch	12,0 cd	12,5 de	11,6 de	10,5 de
Oxyfluorfen (1.8 l ha^−1^)^3^	12,1 cd	12,8 c–e	12,4 bc	11,4 c
Oxyfluorfen (0.9 l ha^−1^)^4^	11,6 de	12,4 e	11,1 e	10,0 f
Hoeing (twice)	12,7 ab	13,6 ab	13,0 a	12,1 ab
Unweeded check	11,1 e	11,7 f	10,2 f	9 g

^1^AE, aqueous extract; ^2^oxyf, oxyfluorfen; ^3^ Oxyfluorfen (1.8 l ha^−1^): the recommended dose of the oxyfluorfen herbicide, ^4^oxyfluorfen (0.9 l ha^−1^): half of the recommended dose of the oxyfluorfen herbicide. Means followed by a letter in common in the same column are not significantly different at 0.05 level of probability according to Duncan multiple range test.

All weeded treatments significantly maintained the total soluble sugars content of onion bulbs during the storage period compared to the unweeded check (Table 6). Total soluble sugars increased by the prolongation of the storage period until 2 months then decreased till the end of the storage (Table 6). The highest values of total soluble sugars content in onion bulbs were obtained from orange processing waste mulch and hoeing treatments without significant differences between them during the storage period (Table 6).

**Table 6:** Effect of weed control methods on total soluble sugars (%) of onion bulbs during storage in season 2018/2019.

Treatments	Storage period (month)			
	0	2	4	6
Orange peel waste AE^1^ 20%	3,72 fg	3,95 ef	3,26 ef	2,63 de
Mango leaves AE 30%	3,83 ef	4,07 de	3,15 f	2,56 ef
Olive oil waste AE 30%	3,60 gh	3,80 f	2,97 g	2,48 f
Orange peel AE 20% + ½oxyf ^2^	4,14 bc	4,54 b	3,72 b	2,94 b
Mango leaves AE 30% + ½oxyf	3,91 de	4,28 b–d	3,48 cd	2,86 b–d
Olive oil waste AE 30% + ½oxyf	3,87 ef	4,25 cd	3,33 de	2,69 c–e
Orange peel waste mulch	4,54 a	4,86 a	4,04 a	3,27 a
Mango leaves mulch	3,97 c–e	4,41 bc	3,54 c	2,85 bc
Olive oil waste mulch	4,00 c–e	4,30 b–d	3,50 cd	2,70 b–d
Rice straw mulch	4,06 b–d	4,37 bc	3,39 c–e	2,66 c–e
Oxyfluorfen (1.8 l ha^−1^)^3^	4,22 b	4,53 b	3,71 b	3,00 b
Oxyfluorfen (0.9 l ha^−1^)^4^	3,54 h	3,74 f	2,93 g	2,43 f
Hoeing (twice)	4,45 a	4,78 a	3,97 a	3,22 a
Unweeded check	2,92 i	3,27 g	2,38 h	1,81 g

^1^AE, aqueous extract; ^2^oxyf, oxyfluorfen; ^3^ Oxyfluorfen (1.8 l ha^−1^): the recommended dose of the oxyfluorfen herbicide, ^4^oxyfluorfen (0.9 l ha^−1^): half of the recommended dose of the oxyfluorfen herbicide. Means followed by a letter in common in the same column are not significantly different at 0.05 level of probability according to Duncan multiple range test.

## Discussion

The results of the current study indicate that weed control treatments can significantly impact the storability of onion bulbs. In this study, OPPW mulch and hoeing treatments recorded the lowest weight loss percentage (Table 1). Petropoulos et al.^20^ stated that water loss is the main limiting factor that determines storage duration, since excessive water losses result in both bulbs increasing weight losses and reducing quality which affect the product marketability. The major factors contributing to weight loss of stored bulbs are respiration, moisture loss, sprouting and handling^21^. The rate of water losses during storage can also be affected by preharvest conditions such as curing method and duration, irrigation regime, fertilization rates^22^, and weed control methods (^9^). The positive effect of all the weed control treatments on reducing weight loss percentage compared to control treatment (Table 1) could be a result of their good performance in controlling weeds in field^10^ thus, reducing weed competition with onion plants for water, light and nutrition. The efficacy of weed control methods on reducing weight loss during storage conditions were also reported in onion^9,18^, and in garlic^19^.

Decay percentage increased during the storage period (Table 2). OPPW mulch and hoeing also were the most effective treatments for reducing decay percentage (Table 2). These results agree with previous work^22,23^ which reported that the quantity of decayed onion bulbs increased with the prolongation of the storage time. The efficacy of different weed control methods on reducing onion bulb decay during storage conditions were also reported in previous research^19,24^. Paltrinieri^25^ mentioned that the decay of vegetables during storage is mostly caused by the infection through mechanical injuries by microorganisms, mostly bacteria and fungi. Furthermore, many vegetables are attacked by decay organisms which penetrate through natural openings or even through the intact skin. These infections may be established during the growth of the plant in the field but remain dormant until after harvest, often becoming visible only during storage. Brewster^26^ indicated that pre- and postharvest curing procedures are critical in the preparation of onion bulbs for long-term storage. Curing is used to produce strong, dry, outer wrapper skins that prevent shrinkage, and to dry the necks of onion bulbs in order to reduce the risk of fungal pathogens infecting fleshy bulb scales during storage^27^.

The increment of bulb dry matter in the first two months of storage (Table 3) could be a result of higher moisture loss rate than respiratory rate, while during 4 to 6 months the respiratory rate was higher than the moisture loss rate. Nabi et al.^22^ and Islam et al.^28^ also recorded the decrease of onion bulb dry matter during storage period. The decline in onion bulb dry matter content is due to metabolic processes inside the living tissue during storage^26^. Sugars are the major component of dry matter and are consumed by respiration^29^. There was a positive correlation between the storage quality and the percentage of dry matter content^30^. A larger content of dry matter means lower water content in the bulbs, reduced metabolic activities in the bulbs, and prolonged longer dormancy period^31^.

Onion bulb firmness was decreased by the prolongation of storage time (Table 4). These results are in agreement with previous work^21,28,32^ which reported a correlation between firmness, bulb dry matter content and TSS content. The decline in onion bulb firmness and dry matter content, is due to metabolic processes inside the living tissue during storage^26^. Onion bulbs contain chemical constituents, such as sugars, protein, and organic acids, as part of the dry matter content, which play a role in regulating the cell turgor pressure and thus influence the firmness of the scales^33^.

The increment of TSS content in the first two months of the storage (Table 5) could be due to moisture loss rate which was higher than respiratory rate, while during the 4 to 6 months, the respiratory rate was higher than the moisture loss rate. This result agrees with previous work^34,35^ which reported a slight increase in TSS at the early stage of storage followed by a reduction to the end of storage period. Similarly, a correlation was also reported between firmness, dry matter content and TSS content of stored onion bulbs^32^. It was suggested that reduction in total soluble content was mainly due to the decrease in total soluble sugar content by transpiration and respiration of bulbs^21^.

The increment of total soluble sugars content (just like total soluble solids) in the first two months of the storage (Table 6) could be due to moisture loss rate which was higher than respiratory rate, while during 4–6 months respiratory rate was higher than the moisture loss rate. This finding agrees with Ilić et al.^36^ who reported slight increase in total soluble sugars at the early stage of storage followed by their reduction. It can also be noticed that total soluble sugars content results of stored onion bulbs in the current study are consistent with the bulb dry matter (Tables 3), firmness (Table 4) and TSS (Table 5). The reason for the high content of TSS and total soluble sugars during the first two months could be attributed to fructans which are important storage polysaccharides in plants. According to Pak et al.^37^, fructans are hydrolyzed to fructose during the initial storage period which leads to high content of TSS and total soluble sugars. However, as the storage dormancy period comes to an end, sprouting starts and sucrose is converted to organic acids and transported for the growth of sprout, ultimately it declines TSS and total soluble sugars content in bulbs^38^.

In summary, well-managed weed control treatments generally promote better crop storability by improving crop health, reducing disease and pest pressure, and minimizing physical damage. However, careful consideration of the type of weed control used is essential to avoid negative effects like chemical residues or unintended damage to the crop.

## Conclusion

It is concluded that the weed control treatments within the onion field study reduced weed infestation within onion plants. Orange peel processing waste showed the highest efficacy in weed control followed by mango leaves and olive oil processing waste. Using the examined agro-industrial wastes as soil mulches or mixing the aqueous extracts with half dose of oxyfluorfen herbicide was more effective in weed control than the sole application of the aqueous extracts. The aqueous extract of orange peel mixed with half dose of oxyfluorfen herbicide was generally the most effective treatment in most of the examined parameters. This treatment could provide an alternative weed control method to hoeing and oxyfluorfen herbicide, especially for regions where manual labor is expensive and unavailable.

The results of the storage experiment revealed that all the examined weed control treatments significantly improved onion storability and maintained better bulb quality compared to the unweeded treatment during a 6-month storage period at room temperature. Orange peel mulch and hoeing treatment were, generally, the most effective preharvest treatments in reducing weight loss and decay percentages and preserving bulb quality.

## Material and methods

### Storage experiment

Storage experiment was carried out at the laboratory of Vegetable Crops Department, Faculty of Agriculture, Cairo University, Giza to evaluate the effect of weed control treatments on quality attributes and storability of onion bulbs during ambient storage. The weed control treatments consisted of aqueous extracts (OPPW20%, OOPW30%, and MLW30%) alone or mixed with half a dose of oxyfluorfen herbicide (½OXYF) (938 ml ha^−1^), soil mulching with orange peel processing waste, mango leaves, olive oil processing waste, and rice straw (OPPWM, MLW, OOPWM, and RSM, respectively), hoeing, oxyfluorfen herbicide (Goal 24% EC) at 938 and 1875 ml (commercial product) ha^−1^, and unweeded check (control treatment).

Onion bulbs were harvested from the treated plots (neck softened) when about 75%-80% of the onion leaves fell and thus became mature. After harvest, bulbs were kept for 14 days in shaded shelter for curing. Then, for storage experiment, bulbs were delivered to the laboratory of Vegetable Crops Department, Faculty of Agriculture, Cairo University, Giza.

A sample of bulbs was taken from each plot and 7 kg of marketable onions was stored in corrugated paper boxes at room temperature (25 ± 5° C and 50% – 60% relative humidity) for 6 months. The experimental design was a complete randomized design with three replications.

### Recorded data

#### Weed control efficacy

During the field experiments weeds were surveyed 70 and 100 DAT and weed samples were randomly collected from one square meter from each experimental unit for estimating weed dry weights. Accordingly, weed control efficacy (WCE) was calculated as follow: WCE (%) = (WDWC − WDWT)/WDWC × 100.

where WDWC is the weed dry weight in weedy check and WDWT is the weed dry weight in treatment.

#### Storage parameters

Samples from each treatment were examined every month for the following properties: weight loss and decay percentages; while dry weight, firmness, total soluble solids, and total soluble sugars were determined at 0, 2, 4 and 6 months of storage. The loss in weight percentage was calculated as follows:

$${\text{Weight loss }}\left( \% \right) = \left( {{\text{W}}_{0} {-}{\text{ W}}_{{\text{n}}} } \right)/{\text{W}}_{0} \times { 1}00$$

where W_o_ = Initial weight (kg), W_n_ = Weight after n days (kg).

The decay percentage was calculated monthly as follows:

$${\text{Decay percentage}} = \left( {{\text{W}}_{{\text{d}}} /{\text{W}}_{0} } \right) \, \times { 1}00$$

where W_d_ = Weight of decayed bulbs (kg), W_o_ = Initial weight (kg).

Measuring Bulb firmness, Total soluble solids, and Total soluble sugars.

Bulb firmness was measured using a Chinese-made digital fruit hardness tester (model Gy-4, Zhejiang Top Cloud-Agri Technology Co., Ltd.). Total soluble solids percentage was determined using a Chinese-made digital refractometer (model TD-45). Total soluble sugars were extracted using 80% ethanol in a hot water path (65 °C) for one hour, then 1 ml of the extract was added to 1 ml of phenol 5% and 5 ml of sulfuric acid^39^. The absorbance was measured at 490 nm using a spectrophotometer (Shimadzu 240 UV/VIS from Japan). Glucose sugar was used to make the calibration curve.

### Statistical analysis

Data for current study were statistically analyzed using M-static version 2.10 and the Duncan multiple range test was used to compare the treatment means^40^. The interactions between treatments and years for all studied variables were non-significant; therefore, data were combined over the two seasons^40^.

## Electronic supplementary material

Below is the link to the electronic supplementary material.

Supplementary Material 1

## Data Availability

The datasets used and/or analysed during the current study available from the corresponding author on reasonable request.
