# Supplementary material for: Efficacy of pre-harvest weed control treatments on onion bulb storability
Source: Sci Rep. 2025 Feb 25;15:6766. doi: 10.1038/s41598-025-89970-6 (PMC11862208; doi:10.1038/s41598-025-89970-6)
Supplement: Supplementary file 1 — Supplementary Material 1 [file 41598_2025_89970_MOESM1_ESM.docx]

**Efficacy of Pre-harvest Weed Control Treatments on Onion Bulb (*Allium cepa* L.) Storability**

Karima F. Abdelgawad^1^, Said A. Shehata^1^, Ibrahim M. El-Metwally^2^,

Ebrahim R. El-Desoki^2^, Kowthar G. El-Rokiek^2^& Fathia A. Elkhawaga^2^

**^1^Vegetable Crops Department, Faculty of Agriculture, Cairo University, Giza, 12613, Egypt**

**^2^Botany Department,** **National Research Centre, Dokki, Cairo, 12622, Egypt**

**Corresponding author:**

[**Fathia_elkhawaga@yahoo.com**](mailto:Fathia_elkhawaga@yahoo.com)

**Table S1. Effect of weed control methods on weight loss percentage of onion bulbs during storage in season 2018/2019.**

| **Treatments** | **Storage period (month)** | | | | | |
| --- | --- | --- | --- | --- | --- | --- |
|  | **1** | **2** | **3** | **4** | **5** | **6** |
| Orange peel waste AE ^1^ 20% | 3.0 bc | 5.6 b-e | 10.1 b | 11.4 d | 13.9 bc | 15.4 cd |
| Mango leaves AE 30% | 3.2 b | 6.1 b-d | 9.2 c | 11.3 d | 13.4 c | 14.8 e |
| Olive oil waste AE 30% | 3.2 b | 5.2 c-e | 7.2 f | 8.6 g | 10.4 g | 11.6 h |
| Orange peel AE 20% + ½oxyf ^2^ | 2.6 cd | 4.6 ef | 6.8 fg | 7.8 h | 9.6 h | 12.5 f |
| Mango leaves AE 30%+ ½oxyf | 2.7 cd | 4.8 de | 6.8 fg | 8.7 g | 10.4 g | 11.8 h |
| Olive oil waste AE 30%+ ½oxyf | 2.6 cd | 5.1 c-e | 7.3 ef | 9.0 g | 10.7 fg | 11.9 gh |
| Orange peel waste mulch | 0.5 f | 3.3 g | 5.7 h | 6.5 i | 9.5 h | 10.7 i |
| Mango leaves mulch | 3.0 bc | 6.3 bc | 8.0 de | 9.6 f | 11.3 e | 12.7 f |
| Olive oil waste mulch | 2.6 cd | 4.7 e | 8.1 d | 9.7 f | 11.2 ef | 12.4 fg |
| Rice straw mulch | 2.4 d | 4.6 ef | 8.6 cd | 10.5 e | 11.9 d | 16.0 b |
| Oxyfluorfen (1.8 l ha^-1^)^3^ | 2.7 cd | 6.4 bc | 8.9 c | 12.2 c | 13.9 bc | 14.9 de |
| Oxyfluorfen (0.9 l ha^-1^)^4^ | 3.0 bc | 6.6 b | 10.2 b | 13.9 b | 14.2 b | 15.5 bc |
| Hoeing (twice) | 1.9 e | 3.4 fg | 6.3 gh | 7.6 h | 9.4 h | 10.9 i |
| Unweeded check | 3.7 a | 8.5 a | 11.4 a | 14.5 a | 17.0 a | 21.9 a |

^1^AE: aqueous extract, ^2^oxyf: oxyfluorfen, ^3^ Oxyfluorfen (1.8 l ha^−1^): the recommended dose of the oxyfluorfen herbicide, ^4^oxyfluorfen (0.9 l ha^-1^): half of the recommended dose of the oxyfluorfen herbicide. Means followed by a letter in common in the same column are not significantly different at 0.05 level of probability according to Duncan multiple range test.

**Table S2. Effect of weed control methods on weight loss percentage of onion bulbs during storage in season 2019/2020.**

| **Treatments** | **Storage period (month)** | | | | | |
| --- | --- | --- | --- | --- | --- | --- |
|  | **1** | **2** | **3** | **4** | **5** | **6** |
| Orange peel waste AE ^1^ 20% | 3.2 e | 6.2 ef | 11.3 c | 14.5 d | 17.1 c | 19.8 e |
| Mango leaves AE 30% | 4.3 d | 7.6 cd | 15.2 b | 17.0 c | 19.8 b | 23.7 b |
| Olive oil waste AE 30% | 4.9 b | 7.8 c | 15.2 b | 17.2 c | 19.7 b | 24.1 b |
| Orange peel AE 20% + ½oxyf ^2^ | 2.0 g | 4.7 gh | 8.4 e | 10.5 gh | 13.3 f | 16.9 g |
| Mango leaves AE 30%+ ½oxyf | 2.4 f | 4.3 h | 10.2 d | 13.3 e | 15.7 d | 17.7 fg |
| Olive oil waste AE 30%+ ½oxyf | 3.3 e | 7.2 c-e | 11.5 c | 14.7 d | 16.2 d | 19.2 e |
| Orange peel waste mulch | 1.5 i | 3.6 h | 6.7 f | 9.4 i | 12.4 g | 15.8 h |
| Mango leaves mulch | 3.5 e | 5.6 fg | 8.4 e | 11.2 g | 14.8 e | 17.4 g |
| Olive oil waste mulch | 4.5 cd | 9.5 b | 11.2 c | 14.7 d | 17.3 c | 20.8 d |
| Rice straw mulch | 3.5 e | 6.7 d-f | 11.2 c | 13.8 e | 16.2 d | 19.8 e |
| Oxyfluorfen (1.8 l ha^-1^)^3^ | 1.9 gh | 6.4 ef | 8.1 e | 12.1 f | 14.8 e | 18.3 f |
| Oxyfluorfen (0.9 l ha^-1^)^4^ | 4.7 bc | 8.3 c | 11.5 c | 18.2 b | 20.3 b | 21.8 c |
| Hoeing (twice) | 1.6 hi | 4.1 h | 7.1 f | 10.0 hi | 12.8 fg | 15.9 h |
| Unweeded check | 5.5 a | 11.8 a | 16.6 a | 19.8 a | 22.4 a | 27.6 a |

^1^AE: aqueous extract, ^2^oxyf: oxyfluorfen, ^3^ Oxyfluorfen (1.8 l ha^−1^): the recommended dose of the oxyfluorfen herbicide, ^4^oxyfluorfen (0.9 l ha^-1^): half of the recommended dose of the oxyfluorfen herbicide. Means followed by a letter in common in the same column are not significantly different at 0.05 level of probability according to Duncan multiple range test.

**Table S3. Effect of weed control methods on decay percentage of onion bulbs during storage in season 2018/2019.**

| **Treatments** | **Storage period (month)** | | | | | |
| --- | --- | --- | --- | --- | --- | --- |
|  | **1** | **2** | **3** | **4** | **5** | **6** |
| Orange peel waste AE ^1^ 20% | 0.0 a | 0.0 b | 0.0 b | 7.4 e | 8.0 de | 8.5 de |
| Mango leaves AE 30% | 0.0 a | 0.0 b | 0.0 b | 9.3 c | 10.0 c | 10.8 c |
| Olive oil waste AE 30% | 0.0 a | 0.0 b | 0.0 b | 9.4 c | 10.5 c | 10.7 c |
| Orange peel AE 20% + ½oxyf ^2^ | 0.0 a | 0.0 b | 0.0 b | 2.5 gh | 2.7 gh | 2.7 h |
| Mango leaves AE 30%+ ½oxyf | 0.0 a | 0.0 b | 0.0 b | 7.6 de | 7.8 de | 7.9 e |
| Olive oil waste AE 30%+ ½oxyf | 0.0 a | 0.0 b | 0.0 b | 8.9 cd | 9.3 cd | 9.5 d |
| Orange peel waste mulch | 0.0 a | 0.0 b | 0.0 b | 0.0 i | 1.0 i | 1.0 i |
| Mango leaves mulch | 0.0 a | 0.0 b | 0.0 b | 3.9 fg | 4.0 fg | 4.2 g |
| Olive oil waste mulch | 0.0 a | 0.0 b | 0.0 b | 4.9 f | 5.4 f | 6.1 f |
| Rice straw mulch | 0.0 a | 0.0 b | 0.0 b | 7.1 e | 7.5 e | 7.9 e |
| Oxyfluorfen (1.8 l ha^-1^)^3^ | 0.0 a | 0.0 b | 0.0 b | 7.4 e | 7.5 e | 7.8 e |
| Oxyfluorfen (0.9 l ha^-1^)^4^ | 0.0 a | 0.0 b | 0.0 b | 12.5 b | 12.5 b | 13.2 b |
| Hoeing (twice) | 0.0 a | 0.0 b | 0.0 b | 1.3 hi | 1.4 hi | 1.6 hi |
| Unweeded check | 0.0 a | 1.2 a | 2.2 a | 14.1 a | 16.6 a | 31.1 a |

^1^AE: aqueous extract, ^2^oxyf: oxyfluorfen, ^3^ Oxyfluorfen (1.8 l ha^−1^): the recommended dose of the oxyfluorfen herbicide, ^4^oxyfluorfen (0.9 l ha^-1^): half of the recommended dose of the oxyfluorfen herbicide. Means followed by a letter in common in the same column are not significantly different at 0.05 level of probability according to Duncan multiple range test.

**Table S4. Effect of weed control methods on decay percentage of onion bulbs** **during storage in season 2019/2020.**

| **Treatments** | **Storage period (month)** | | | | | |
| --- | --- | --- | --- | --- | --- | --- |
|  | **1** | **2** | **3** | **4** | **5** | **6** |
| Orange peel waste AE ^1^ 20% | 3.3 d | 3.9 e | 7.3 e | 10.8 c | 11.9 c | 13.6 c |
| Mango leaves AE 30% | 3.4 d | 5.1 d | 10.2 b | 10.7 c | 12.1 bc | 14.3 b |
| Olive oil waste AE 30% | 3.9 bc | 6.5 b | 9.6 c | 11.5 b | 12.7 b | 14.5 b |
| Orange peel AE 20% + ½oxyf ^2^ | 1.5 f | 4.1 e | 4.1 i | 6.7 g | 8.1 f | 12.0 d |
| Mango leaves AE 30%+ ½oxyf | 3.5 cd | 5.1 d | 7.1 ef | 9.8 d | 11.1 d | 11.6 de |
| Olive oil waste AE 30%+ ½oxyf | 4.1 b | 6.1 bc | 9.6 c | 10.9 e | 11.6 c | 14.2 bc |
| Orange peel waste mulch | 0.8 g | 1.7 g | 5.0 h | 5.0 i | 5.4 g | 7.2 h |
| Mango leaves mulch | 1.5 f | 4.9 d | 7.0 ef | 8.1 f | 9.0 e | 11.0 ef |
| Olive oil waste mulch | 2.0 e | 5.3 d | 8.1 d | 9.7 d | 11.5 cd | 11.8 d |
| Rice straw mulch | 2.1 e | 5.5 cd | 7.8 d | 9.2 e | 11.4 cd | 11.8 d |
| Oxyfluorfen (1.8 l ha^-1^)^3^ | 1.5 f | 3.0 f | 6.6 fg | 8.4 f | 8.4 ef | 10.5 f |
| Oxyfluorfen (0.9 l ha^-1^)^4^ | 4.2 b | 6.1 bc | 9.4 c | 11.5 b | 12.1 bc | 14.6 b |
| Hoeing (twice) | 0.9 g | 2.1 g | 6.0 g | 6.0 h | 6.0 g | 8.6 g |
| Unweeded check | 5.4 a | 7.8 a | 11.1 a | 12.5 a | 14.1 a | 15.7 a |

^1^AE: aqueous extract, ^2^oxyf: oxyfluorfen, ^3^ Oxyfluorfen (1.8 l ha^−1^): the recommended dose of the oxyfluorfen herbicide, ^4^oxyfluorfen (0.9 l ha^-1^): half of the recommended dose of the oxyfluorfen herbicide. Means followed by a letter in common in the same column are not significantly different at 0.05 level of probability according to Duncan multiple range test.

**Table S5. Effect of weed control methods on dry matter (%) of onion bulbs during storage in season 2018/2019.**

| **Treatments** | **Storage period (month)** | | | |  |
| --- | --- | --- | --- | --- | --- |
|  | **0** | **2** | **4** | **6** | |
| Orange peel waste AE ^1^ 20% | 12.1 bc | 15.6 de | 15.0 ef | 14.7 g | |
| Mango leaves AE 30% | 10.9 ef | 14.9 ef | 14.7 ef | 14.1 h | |
| Olive oil waste AE 30% | 10.4 f | 16.9 d | 15.9 e | 15.2 fg | |
| Orange peel AE 20% + ½oxyf ^2^ | 11.8 bc | 20.8 ab | 19.5 bc | 19.3 b | |
| Mango leaves AE 30%+ ½oxyf | 11.6 cd | 19.0 c | 18.5 cd | 18.0 c | |
| Olive oil waste AE 30%+ ½oxyf | 11.9 bc | 19.5 bc | 17.6 d | 15.4 ef | |
| Orange peel waste mulch | 12.4 ab | 21.4 a | 21.2 a | 20.9 a | |
| Mango leaves mulch | 11.0 d-f | 21.1 a | 18.4 cd | 15.9 de | |
| Olive oil waste mulch | 11.2 de | 16.9 d | 15.5 ef | 14.9 fg | |
| Rice straw mulch | 11.1 de | 16.7 d | 15.0 ef | 12.9 i | |
| Oxyfluorfen (1.8 l ha^-1^)^3^ | 12.0 bc | 16.4 de | 16.1 e | 16.0 d | |
| Oxyfluorfen (0.9 l ha^-1^)^4^ | 11.0 d-f | 15.6 de | 14.2 f | 12.8 i | |
| Hoeing (twice) | 12.8 a | 20.4 a-c | 19.5 bc | 19.1 b | |
| Unweeded check | 11.1 g | 13.8 f | 12.6 g | 12.1 j | |

^1^AE: aqueous extract, ^2^oxyf: oxyfluorfen, ^3^ Oxyfluorfen (1.8 l ha^−1^): the recommended dose of the oxyfluorfen herbicide, ^4^oxyfluorfen (0.9 l ha^-1^): half of the recommended dose of the oxyfluorfen herbicide. Means followed by a letter in common in the same column are not significantly different at 0.05 level of probability according to Duncan multiple range test.

**Table S6. Effect of weed control methods on dry matter (%) of onion bulbs during storage months in season 2019/2020.**

| **Treatments** | **Storage period (month)** | | | |  |
| --- | --- | --- | --- | --- | --- |
|  | **0** | **2** | **4** | **6** | |
| Orange peel waste AE ^1^ 20% | 10.3 cd | 20.6 e | 18.9 cd | 18.5 d-f | |
| Mango leaves AE 30% | 10.3 cd | 20.5 e | 18.8 cd | 17.9 e-g | |
| Olive oil waste AE 30% | 9.4 e | 20.3 e | 18.6 d | 17.5 fg | |
| Orange peel AE 20% + ½oxyf ^2^ | 10.1 d | 23.4 ab | 22.1 a | 21.3 a-c | |
| Mango leaves AE 30%+ ½oxyf | 10.7 bc | 21.5 d | 20.9 b | 20.2 b-d | |
| Olive oil waste AE 30%+ ½oxyf | 11.0 b | 21.5 d | 19.8 b-d | 18.8 d-f | |
| Orange peel waste mulch | 11.4 a | 23.9 a | 23.1 a | 22.8 a | |
| Mango leaves mulch | 10.2 d | 21.6 cd | 20.3 b | 19.6 c-e | |
| Olive oil waste mulch | 10.3 cd | 21.4 d | 20.1 bc | 18.1 e-g | |
| Rice straw mulch | 10.4 cd | 23.2 b | 20 bc | 18.5 d-f | |
| Oxyfluorfen (1.8 l ha^-1^)^3^ | 9.1 e | 20.3 e | 17.3 e | 16.5 g | |
| Oxyfluorfen (0.9 l ha^-1^)^4^ | 9.3 e | 18.1 f | 15.5 f | 13.4 h | |
| Hoeing (twice) | 10.4 cd | 23.4 ab | 22.7 a | 21.5 ab | |
| Unweeded check | 6.8 f | 15.1 g | 12.9 g | 10.7 i | |

^1^AE: aqueous extract, ^2^oxyf: oxyfluorfen, ^3^ Oxyfluorfen (1.8 l ha^−1^): the recommended dose of the oxyfluorfen herbicide, ^4^oxyfluorfen (0.9 l ha^-1^): half of the recommended dose of the oxyfluorfen herbicide. Means followed by a letter in common in the same column are not significantly different at 0.05 level of probability according to Duncan multiple range test.

**Table S7. Effect of weed control methods on firmness (kg/cm^2^) of onion bulbs during storage in season 2018/2019.**

| **Treatments** | **Storage period (month)** | | | |  |
| --- | --- | --- | --- | --- | --- |
|  | **0** | **2** | **4** | **6** | |
| Orange peel waste AE ^1^ 20% | 12.6 c-e | 11.1 cd | 10.2 fg | 10.0 d | |
| Mango leaves AE 30% | 12.0 c-f | 11.2 cd | 10.2 fg | 9.7 de | |
| Olive oil waste AE 30% | 10.8 fg | 10.4 d | 9.8 g | 9.2 e | |
| Orange peel AE 20% + ½oxyf ^2^ | 13.0 cd | 12.6 b | 12.2 b | 11.9 b | |
| Mango leaves AE 30%+ ½oxyf | 13.2 c | 12.0 bc | 11.5 c | 11.1 c | |
| Olive oil waste AE 30%+ ½oxyf | 11.7 d-f | 10.9 cd | 10.6 d-f | 10.1 d | |
| Orange peel waste mulch | 15.8 a | 14.8 a | 13.3 a | 12.5 a | |
| Mango leaves mulch | 12.4 c-e | 12.0 bc | 11.5 c | 11.2 c | |
| Olive oil waste mulch | 11.7 d-f | 11.1 cd | 10.3 fg | 10.0 d | |
| Rice straw mulch | 12.1 c-f | 11.5 b-d | 11.1 cd | 11.0 c | |
| Oxyfluorfen (1.8 l ha^-1^)^3^ | 11.6 ef | 11.3 b-d | 11.0 c-e | 10.8 c | |
| Oxyfluorfen (0.9 l ha^-1^)^4^ | 11.5 ef | 10.9 cd | 10.5 ef | 10.0 d | |
| Hoeing (twice) | 14.5 b | 14.0 a | 12.5 b | 12.0 ab | |
| Unweeded check | 9.8 g | 9.1 e | 8.5 h | 8.0 f | |

^1^AE: aqueous extract, ^2^oxyf: oxyfluorfen, ^3^ Oxyfluorfen (1.8 l ha^−1^): the recommended dose of the oxyfluorfen herbicide, ^4^oxyfluorfen (0.9 l ha^-1^): half of the recommended dose of the oxyfluorfen herbicide. Means followed by a letter in common in the same column are not significantly different at 0.05 level of probability according to Duncan multiple range test.

**Table S8. Effect of weed control methods on firmness (kg/cm^2^) of onion bulbs during storage in season 2019/2020.**

| **Treatments** | **Storage period (month)** | | | |  |
| --- | --- | --- | --- | --- | --- |
|  | **0** | **2** | **4** | **6** | |
| Orange peel waste AE ^1^ 20% | 12.3 f | 10.3 f | 9.4 h | 9.1 fg | |
| Mango leaves AE 30% | 12.2 f | 9.3 g | 9.3 h | 9 g | |
| Olive oil waste AE 30% | 11.1 g | 9.9 g | 9.3 h | 9.1 fg | |
| Orange peel AE 20% + ½oxyf ^2^ | 14.8 c | 12.8 c | 11.0 ef | 10.5 d | |
| Mango leaves AE 30%+ ½oxyf | 15.3 bc | 12.9 c | 12.5 d | 11.9 c | |
| Olive oil waste AE 30%+ ½oxyf | 14.2 d | 11.7 e | 11.1 e | 10.6 d | |
| Orange peel waste mulch | 16.1 a | 15.6 a | 14.5 a | 13.9 a | |
| Mango leaves mulch | 14.9 c | 12.4 cd | 11.1 e | 10.3 d | |
| Olive oil waste mulch | 13.5 e | 11.5 e | 10.8 ef | 10.3 d | |
| Rice straw mulch | 15.1 c | 12.0 de | 10.2 g | 9.6 ef | |
| Oxyfluorfen (1.8 l ha^-1^)^3^ | 15.8 ab | 12.8 c | 13.1 c | 12.7 b | |
| Oxyfluorfen (0.9 l ha^-1^)^4^ | 13.8 de | 11.6 e | 10.5 fg | 10.1 de | |
| Hoeing (twice) | 15.7 ab | 14.6 b | 13.9 b | 12.7 b | |
| Unweeded check | 10.4 h | 7.9 h | 7.5 i | 7.1 h | |

^1^AE: aqueous extract, ^2^oxyf: oxyfluorfen, ^3^ Oxyfluorfen (1.8 l ha^−1^): the recommended dose of the oxyfluorfen herbicide, ^4^oxyfluorfen (0.9 l ha^-1^): half of the recommended dose of the oxyfluorfen herbicide. Means followed by a letter in common in the same column are not significantly different at 0.05 level of probability according to Duncan multiple range test.

**Table S9. Effect of weed control methods on total soluble solids (%) of onion bulbs during storage in season 2018/2019.**

| **Treatments** | **Storage period (month)** | | | |  |
| --- | --- | --- | --- | --- | --- |
|  | **0** | **2** | **4** | **6** | |
| Orange peel waste AE ^1^ 20% | 13.5 b-d | 14.5 bc | 11.5 e | 10.0 g | |
| Mango leaves AE 30% | 13.5 b-d | 14.4 bc | 12.3 d | 10.6 f | |
| Olive oil waste AE 30% | 12.6 f | 14.5 bc | 12.2 d | 11.5 de | |
| Orange peel AE 20% + ½oxyf ^2^ | 13.6 a-c | 14.4 bc | 13.6 b | 12.5 c | |
| Mango leaves AE 30%+ ½oxyf | 13.0 d-f | 14.6 b | 13.7 b | 12.8 bc | |
| Olive oil waste AE 30%+ ½oxyf | 13.1 c-f | 14.5 bc | 13.1 c | 12.5 c | |
| Orange peel waste mulch | 14.1 a | 15.3 a | 14.4 a | 13.4 a | |
| Mango leaves mulch | 12.9 d-f | 14.6 b | 13.6 b | 11.1 ef | |
| Olive oil waste mulch | 13.0 d-f | 14.1 b-d | 13.6 b | 12.5 c | |
| Rice straw mulch | 13.0 d-f | 13.6 de | 12.3 d | 11.0 ef | |
| Oxyfluorfen (1.8 l ha^-1^)^3^ | 13.0 b-e | 14.4 bc | 13.7 b | 11.8 d | |
| Oxyfluorfen (0.9 l ha^-1^)^4^ | 12.8 ef | 13.9 cd | 12.0 d | 10.7 f | |
| Hoeing (twice) | 13.7 ab | 15.3 a | 14.3 a | 13.1 ab | |
| Unweeded check | 12.0 g | 13.1 e | 10.6 f | 9.4 h | |

^1^AE: aqueous extract, ^2^oxyf: oxyfluorfen, ^3^ Oxyfluorfen (1.8 l ha^−1^): the recommended dose of the oxyfluorfen herbicide, ^4^oxyfluorfen (0.9 l ha^-1^): half of the recommended dose of the oxyfluorfen herbicide. Means followed by a letter in common in the same column are not significantly different at 0.05 level of probability according to Duncan multiple range test.

**Table S10. Effect of weed control methods on total soluble solids (%) of onion bulbs during storage in season 2019/2020.**

| **Treatments** | **Storage period (month)** | | | |  |
| --- | --- | --- | --- | --- | --- |
|  | **0** | **2** | **4** | **6** | |
| Orange peel waste AE ^1^ 20% | 11.1 c | 11.3 cd | 11.0 c | 9.4 e | |
| Mango leaves AE 30% | 10.8 cd | 11.2 de | 10.5 de | 9.1 f | |
| Olive oil waste AE 30% | 10.8 cd | 10.9 e | 10.5 de | 9.3 ef | |
| Orange peel AE 20% + ½oxyf ^2^ | 11.0 cd | 11.4 cd | 11.2 bc | 10.4 c | |
| Mango leaves AE 30%+ ½oxyf | 11.2 bc | 11.7 bc | 11.2 bc | 10.8 b | |
| Olive oil waste AE 30%+ ½oxyf | 11.0 cd | 11.3 cd | 10.6 de | 10.5 c | |
| Orange peel waste mulch | 12.0 a | 12.1 a | 11.8 a | 11.2 a | |
| Mango leaves mulch | 10.8 cd | 11.4 cd | 11.1 c | 10.4 c | |
| Olive oil waste mulch | 10.8 cd | 11.3 cd | 11.0 c | 10.5 c | |
| Rice straw mulch | 10.9 cd | 11.3 cd | 10.8 cd | 9.9 d | |
| Oxyfluorfen (1.8 l ha^-1^)^3^ | 11.1 c | 11.2 de | 11.0 c | 10.9 b | |
| Oxyfluorfen (0.9 l ha^-1^)^4^ | 10.4 de | 10.9 e | 10.2 ef | 9.3 ef | |
| Hoeing (twice) | 11.7 ab | 11.8 ab | 11.6 ab | 11.0 ab | |
| Unweeded check | 10.2 e | 10.3 f | 9.8 f | 8.6 g | |

^1^AE: aqueous extract, ^2^oxyf: oxyfluorfen, ^3^ Oxyfluorfen (1.8 l ha^−1^): the recommended dose of the oxyfluorfen herbicide, ^4^oxyfluorfen (0.9 l ha^-1^): half of the recommended dose of the oxyfluorfen herbicide. Means followed by a letter in common in the same column are not significantly different at 0.05 level of probability according to Duncan multiple range test.

**Table S11. Effect of weed control methods on total soluble sugars (%) of onion bulbs during storage in season 2018/2019.**

| **Treatments** | **Storage period (month)** | | | |  |
| --- | --- | --- | --- | --- | --- |
|  | **0** | **2** | **4** | **6** | |
| Orange peel waste AE ^1^ 20% | 3.43 c-f | 3.78 de | 2.87 de | 2.06 cd | |
| Mango leaves AE 30% | 3.36 d-f | 3.75 d-f | 2.81 de | 2.11 c | |
| Olive oil waste AE 30% | 3.20 f | 3.51 f | 2.50 f | 1.84 e | |
| Orange peel AE 20% + ½oxyf ^2^ | 3.70 b | 4.31 b | 3.22 bc | 2..31 b | |
| Mango leaves AE 30%+ ½oxyf | 3.31 ef | 3.91 cd | 2.87 de | 2.19 bc | |
| Olive oil waste AE 30%+ ½oxyf | 3.41 c-f | 3.90 cd | 2.65 ef | 1.90 de | |
| Orange peel waste mulch | 4.18 a | 4.66 a | 3.41 ab | 2.72 a | |
| Mango leaves mulch | 3.55 b-e | 4.14 bc | 2.97 cd | 2.31 b | |
| Olive oil waste mulch | 3.63 bc | 4.08 bc | 2.97 cd | 2.18 bc | |
| Rice straw mulch | 3.61 b-d | 4.11 bc | 2.86 de | 2.07 cd | |
| Oxyfluorfen (1.8 l ha^-1^)^3^ | 3.78 b | 4.25 b | 3.03 cd | 2.22 bc | |
| Oxyfluorfen (0.9 l ha^-1^)^4^ | 3.26 f | 3.54 ef | 2.47 f | 1.86 e | |
| Hoeing (twice) | 4.10 a | 4.56 a | 3.47 a | 2.64 a | |
| Unweeded check | 2.74 g | 3.26 g | 2.06 g | 1.45 f | |

^1^AE: aqueous extract, ^2^oxyf: oxyfluorfen, ^3^ Oxyfluorfen (1.8 l ha^−1^): the recommended dose of the oxyfluorfen herbicide, ^4^oxyfluorfen (0.9 l ha^-1^): half of the recommended dose of the oxyfluorfen herbicide. Means followed by a letter in common in the same column are not significantly different at 0.05 level of probability according to Duncan multiple range test.

**Table S12. Effect of weed control methods on total soluble sugars (%) of onion bulbs during storage in season 2019/2020.**

| **Treatments** | **Storage period (month)** | | | |  |
| --- | --- | --- | --- | --- | --- |
|  | **0** | **2** | **4** | **6** | |
| Orange peel waste AE ^1^ 20% | 4.01 f | 4.11 f | 3.65 f | 3.19 d | |
| Mango leaves AE 30% | 4.30 e | 4.39 e | 3.48 g | 3.01 e | |
| Olive oil waste AE 30% | 3.99 f | 4.08 fg | 3.44 g | 3.11 de | |
| Orange peel AE 20% + ½oxyf ^2^ | 4.58 b-d | 4.77 bc | 4.22 c | 3.56 b | |
| Mango leaves AE 30%+ ½oxyf | 4.51 c-e | 4.65 b-d | 4.09 c-e | 3.53 b | |
| Olive oil waste AE 30%+ ½oxyf | 4.32 e | 4.59 cd | 4.01 de | 3.48 b | |
| Orange peel waste mulch | 4.89 a | 5.06 a | 4.67 a | 3.81 a | |
| Mango leaves mulch | 4.38 de | 4.67 b-d | 4.11 cd | 3.38 bc | |
| Olive oil waste mulch | 4.37 de | 4.51 de | 4.03 de | 3.22 cd | |
| Rice straw mulch | 4.51 c-e | 4.63 b-d | 3.92 e | 3.25 cd | |
| Oxyfluorfen (1.8 l ha^-1^)^3^ | 4.66 bc | 4.81 b | 4.39 b | 3.77 a | |
| Oxyfluorfen (0.9 l ha^-1^)^4^ | 3.81 f | 3.93 g | 3.39 g | 3.00 e | |
| Hoeing (twice) | 4.79 ab | 4.99 a | 4.46 b | 3.79 a | |
| Unweeded check | 3.09 g | 3.27 h | 2.69 h | 2.16 f | |

^1^AE: aqueous extract, ^2^oxyf: oxyfluorfen, ^3^ Oxyfluorfen (1.8 l ha^−1^): the recommended dose of the oxyfluorfen herbicide, ^4^oxyfluorfen (0.9 l ha^-1^): half of the recommended dose of the oxyfluorfen herbicide. Means followed by a letter in common in the same column are not significantly different at 0.05 level of probability according to Duncan multiple range test.
